# Effects of Household Air Pollution (HAP) on Cardiovascular Diseases in Low- and Middle-Income Countries (LMICs): A Systematic Review and Meta-Analysis

**DOI:** 10.3390/ijerph19159298

**Published:** 2022-07-29

**Authors:** Akorede Adekoya, Sudhir K. Tyagi, Christiana N. Duru, Imran Satia, Vibhu Paudyal, Om P. Kurmi

**Affiliations:** 1 Global Health, McMaster University, Hamilton, ON L8S 4L8, Canada; akoredeadekoya.f@gmail.com; 2 Department of Mechanical Engineering, Indian Institute of Technology Delhi, Hauz Khas, New Delhi 110016, India; sudhirtyagi@yahoo.com; 3 NES Healthcare, Aylesbury HP20 1SE, UK; christianaduru@priorygroup.com; 4 Department of Medicine, McMaster University, Hamilton, ON L8S 4L8, Canada; satiai@mcmaster.ca; 5 Firestone Institute for Respiratory Health, St Joseph’s Healthcare, Hamilton, ON L8S 4L8, Canada; 6 Department of Health Research Methods, Evidence, and Impact, Faculty of Health Sciences, McMaster University, Hamilton, ON L8S 4L8, Canada; 7 McMaster Institute for Research on Ageing, McMaster University, Hamilton, ON L8S 4L8, Canada; 8 School of Pharmacy, College of Medical and Dental Sciences, Sir Robert Aitken Institute for Medical Research, University of Birmingham, Birmingham B15 2TT, UK; 9 Centre for Intelligent Healthcare, Institute of Health and Wellbeing, Richard Cross Building, Coventry University, Coventry CV1 5FB, UK; 10 Nexus Institute of Research and Innovation, Lalitpur 44700, Nepal

**Keywords:** household air pollution, cardiovascular disease, particulate matter, biomass fuel, low-and middle-income countries, hypertension

## Abstract

Background: Out of over 3 billion people exposed to household air pollution (HAP), approximately 4 million die prematurely, most from cardiorespiratory diseases. Although many recent studies have reported adverse effects of HAP on cardiovascular outcomes, the findings are inconsistent. Objectives: The primary aim of this systematic review is to critically appraise the published studies and report the pooled summary of the findings on the association between HAP and cardiovascular outcomes, particularly in LMICs. Methods: During this systematic review and meta-analysis, six databases were searched systematically, and the protocol was published in PROSPERO (CRD 42021248800). Only peer-reviewed English-language studies published from 1980 to March 2021 were included. We extracted data for the population ≥ 18 years old. Newcastle–Ottawa Criteria were used to assess the quality of evidence. The heterogeneity and publication bias of the studies was evaluated. A meta-analysis was conducted using a random-effect model to pool the findings from published studies. Results: In sixteen studies totaling 547,463 cases, 319,180 were exposed to HAP. The pooled estimate suggested an overall 13% higher risk of CVDs, and a 21% higher risk of CVD mortality in LMICs among those exposed to HAP. Similarly, the increased risk of stroke and cerebrovascular accidents, heart failure, and hypertension was statistically significant among those exposed to HAP but not with myocardial infarction, IHD, eclampsia/preeclampsia, and carotid intima-media thickness. Conclusions: Our findings suggest exposure to HAP increases the risk of cardiovascular outcomes.

## 1. Introduction

Air pollution is a growing public health problem in low- and middle-income countries (LMICs) [1]. Over 3 billion people worldwide cook using open fires or simple stoves fueled by kerosene, biomass (wood, crop waste, and dung), and coal [2]. Thus, they are exposed to a high concentration of household air pollution (HAP) generated within household settings, including for heating [3]. In LMICs, particularly rural populations, about 90% of the population rely on solid fuels and kerosene as primary domestic energy sources [4]. The fuel combustion process produces high levels of HAP with a range of dangerous pollutants that penetrate deeply into the human body systems and result in several chronic diseases [5].

The exposure to the HAP concentration depends upon many factors, including the types of fuel, moisture content, types of stoves, combustion efficiency, and ventilation [6].

Globally, cardiovascular disease (CVD)-related death is the number of causes of mortality, accounting for approximately 18.6 million premature deaths in 2019 [1]. Although smoking and dietary factors are the major risk factors for cardiovascular morbidity and mortality, exposure to air pollution has been reported to be number three and, in some LMICs, reported to be number one by the global burden of diseases study. Annually, about 4 million people die prematurely from illnesses, particularly cardiovascular diseases, attributed to HAP [1]. Over the last 5–6 years, many new epidemiological studies have reported an association between the harmful effects of HAPs on both acute and chronic cardiovascular health similar to that reported earlier for ambient air pollution [2,7,8]. The risk estimated by many studies on the effect of HAP and CVDs varies widely depending upon the population group, geographical regions, study types, and factors taken into account during the analysis. Studies on the effects of HAP and CVDs in LMICs have been growing. Therefore, it is important to review and summarize the findings from the published studies using a robust technique. We, therefore, planned to systematically review all the studies for their methodical rigorousness and summarize the relationship between HAP and CVDs. The primary aim of this study is to investigate the associations between HAP exposure and the risk of CVDs through systematic review and meta-analysis.

## 2. Methods

This systematic review and meta-analysis study was performed in accordance with the guidelines of the Preferred Reporting Items for Systematic Reviews and Meta-Analyses (PRISMA). The protocol was published in PROSPERO (CRD 42021248800).

### 2.1. Search Strategy

Database research was performed on 24 and 25 March 2021. This review sought to identify all related papers in peer-reviewed journals on the relationship of household air pollution to cardiovascular diseases. Six databases (PubMed, ProQuest, Web of Science, Ovid Embase, Global Health, and Latin American and Caribbean Literature (LILAC)) were searched for published studies. In addition to the electronic search, the reference lists of the selected papers were manually searched for relevant literature that could be added to the study; this was done to capture all available articles pertinent to this study. Only literature published from 1980 to the date of search (25 March 2021) was considered in this study. The following keywords were used for searching: household air pollution, cardiovascular diseases, particulate matter, biomass fuel, and low- and middle-income countries. Low- and middle-income countries were classified using the World Bank classification of countries based on national estimates of gross national income (GNI) per capita for the previous year (https://www.datatopics.worldbank.org/world-development-indicators/the-world-by-income-and-region.html (accessed on 24 March 2021)). The connectors ‘AND’ and ‘OR’ were used to link search terms. The potentially relevant references with their abstracts were exported into Covidence, and after the removal of duplicates, the titles and abstracts were screened to eliminate unrelated papers. The full texts of each paper were reviewed by two co-authors independently, and those that met the inclusion criteria were obtained, abstracted, and summarized using a standardized format.

### 2.2. Inclusion and Exclusion Criteria

#### 2.2.1. Population

The target population for this study was people older than 18 and exposed to HAP in LMIC settings. Studies conducted on animals, children under 18 years, or focused on participants exposed to indoor air pollution from occupation sources were excluded.

#### 2.2.2. Exposure

This study defined exposure as those exposed to household air pollution arising from indoor cooking or heating using domestic biomass fuels. We did not limit the studies based on the duration of exposure. The exposure of interest was HAP, generated from indoor cooking, heating, and lighting only using biomass fuel and/or coal. Studies that quantified exposure through direct measurement of specific pollutants, questionnaires regarding exposure history, comparison of groups exposed to types of exposure (e.g., different types), or before and after an intervention to reduce exposure were included. Studies that focus on ambient air pollution, occupational exposures, tobacco smoke exposure, allergens, non-fuel combustion or non-solid fuels sources, or studies that did not distinguish between ambient and household air pollution were not considered in the selection process.

#### 2.2.3. Comparator

There was no restriction on the type of comparator for fuel used in the included studies. Studies that lacked a comparator group, or those that did not report any outcome of interest (fuel), were excluded.

### 2.3. Outcomes

The outcomes considered in this study indicated cardiovascular health, such as coronary artery disease, heart failure, thrombosis, stroke, heart attack, myocardial infarction, peripheral arterial disease, and other cardiovascular diseases, previously associated with HAP exposure. Cardiovascular death and hospital emergency/admission were also considered. The study’s major end-point of interest was the connections between HAP and cardiovascular diseases. No limit was placed on the follow-up duration of outcome measures. Studies that reported non-indoor air quality and focused only on fuel utilization, cooking time, and climate were excluded.

### 2.4. Study Designs

No limit was placed on the types of study designs. All randomized control trials (RCTs and quasi-RCTs), non-randomized control trials (in the form of cohort, case–control, and cross-sectional studies), and time-series studies conducted in LMICs countries were included in this study. Only peer-reviewed studies, whose full texts are available, were included in the selected studies. In addition, only articles written in the English language were deemed eligible for this study.

### 2.5. Selection Process

**Figure 1:**
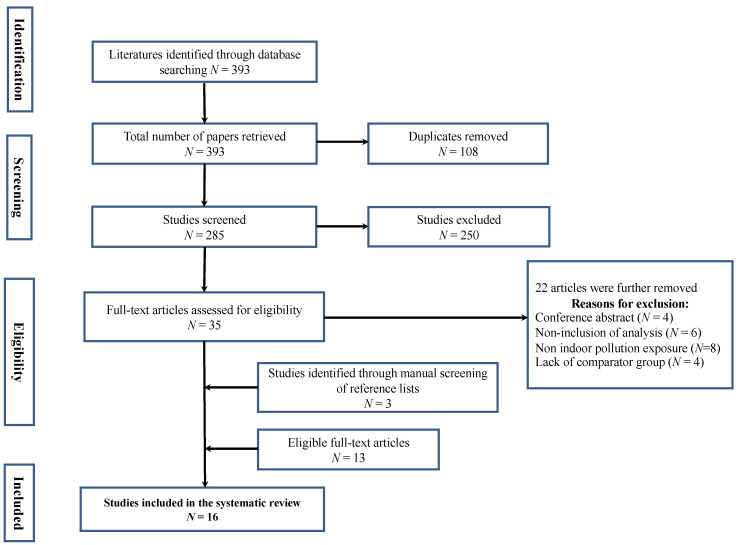
The PRISMA flow diagram for the selection of studies for the systematic review.

After removing duplicates, relevant articles were initially determined by titles and abstracts, followed by retrieval and assessment of full-text articles. Two reviewers (A.A.) and (N.D.) independently conducted the article selection using the eligibility criteria. Reasons for exclusion were noted at each stage of the selection process. Differences in opinions between the two reviewers (A.A. and N.D.) on the selection process were discussed with a third independent reviewer (O.K.), who adjudicated the matter. The selection process is illustrated in Figure 1 using a PRISMA flow diagram.

### 2.6. Data Extraction

From the selected studies, the extracted data included *study characteristics* (title of the article, author(s), name(s), year of publication, geographical setting, features of the target population, sample size, study design, region, age group, single or multipollutant model, and types of cardiovascular disease events), *exposure details* (the kind of pollutants from household air pollution that the participants were exposed to, length of exposure, types of air pollution measurements, length of measurement, and equipment used for measurement, and results, if any), and *health outcomes* (end-point outcomes, measurement methods, method appropriateness, time point measured, confounders included in the analysis, and significant findings, etc.)

### 2.7. Quality Assessment

Two individuals independently assessed the quality of the selected studies. The Newcastle–Ottawa Scale was used to rate the design, validity, reliability, generalizability, and risk of bias of the articles selected for review. This method has been used previously in other systematic reviews [9].

### 2.8. Statistical Analyses

The pooled summary of odds ratios (OR) was computed by pooling the OR and its 95% confidence intervals (CI) from all the relevant papers to calculate the log odds ratio and standard error. The summary correlation and 95% CI were computed by pooling each relevant paper’s correlation and sample size. The fixed-effect model was implemented when a fixed population effect size was assumed; otherwise, the random-effects model was used. Statistical heterogeneity was assessed using the Higgins *I*^2^ statistic, at the value of >50%, and the Cochrane Q (χ^2^ test), at the value of *p* <  0.10 [10]. To account for this heterogeneity, the random-effects model was employed. Publication bias was assumed in the presence of an asymmetrical funnel plot and based on Egger’s regression test (*p*-value < 0.10). Duvall and Tweedie’s trim and fill method was used if publication bias was established [11]. Subgroup analysis was conducted based on household air pollutants and cardiovascular disease types. Data analysis was performed using Review Manager version 5.4 (the Nordic Cochrane Centre, the Cochrane Collaboration, Copenhagen, Denmark) and Comprehensive Meta-Analysis v3 software [12]. Significance was established at values of *p*  <  0.05.

## 3. Results

### 3.1. Search Results

The initial electronic literature search process retrieved 393 studies. After removing 108 duplicates, 285 titles and abstracts were screened. From these, 250 were eliminated for failing to meet the eligibility criteria. The excluded articles did not include either household air pollution exposure or an outcome of cardiovascular diseases. Next, 22 articles were further removed because they were unrelated to the study. Finally, three additional studies found through manual screening of selected studies met the inclusion criteria and were added to the selected articles. Sixteen articles were included in this systematic review study (Figure 1).

### 3.2. Characteristics of Selected Studies

The major characteristics of each selected study are presented in Table 1. The 16 articles selected for this study showcase multiple design protocols: nine were cross-sectional studies, six were prospective cohort studies, and one was a case–control study. The studies were conducted in different nations: six in China, four in India, and one in Bangladesh, Thailand, Peru, Ghana, Iran, and Nepal. Most studies were based in rural settings and recruited participants from communities. Most of the studies compared the use of biomass fuels with non-use or cleaner fuels. As reported by five papers, the primary source of household air pollutants was wood. This was followed by solid fuel exposure and charcoal use, documented in four papers. One of the studies considered exposure to PM at the concentration of 2.5 ppm, while another examined association with exposure to indoor incense burning. The selected studies examined associations with various cardiovascular and cardiorespiratory diseases. The diseases concerned and the number of studies that assessed the relationships between HAP and health conditions are as follows: carotid artery intima-media thickness (CIMT) (*n* = 2), systolic blood pressure SBP (*n* = 2), diastolic blood pressure DBP (*n* = 2), hypertension (HTN) (*n* = 3), eclampsia/pre-eclampsia (*n* = 3), IHD (*n* = 3), stroke or cerebrovascular accident (*n* = 5), and cardiovascular-related mortality (*n* = 6). The potential presence of confounding factors was addressed using various methods such as restriction, matching, and statistical adjustments. Some of the selected studies have important limitations, as discussed below.

### 3.3. Cardiovascular Risk and Diseases Examined in Selected Studies

Two cross-sectional studies examined the associations between exposure to smoke from biomass fuels compared with clean fuels (gas or electricity) and the risk of atherosclerosis as measured by carotid intima-media thickness (CIMT) [13,15]. The sample size for these studies was relatively small; they reported significantly higher mean CIMT (0.66 mm and 0.75 mm) for biomass fuel users compared to clean fuel, respectively. One of the studies exposed a random subset of participants to 24 h of indoor particulate matter PM_2.5_ concentration [10]. The result showed that the median 24 h concentration of indoor PM was significantly higher in biomass fuel users (280 μg/m^3^) compared to clean fuel users (14 μg/m^3^) [13].

Two studies assessed the effects of biomass fuel exposure on systolic blood pressure (SBP) and diastolic blood pressure (DBP) [14,23]. Wylie et al. reported that pregnant women using wood as fuel have lower diastolic blood pressure at delivery than clean fuels [14]. In contrast, Arku et al. reported no significant difference between the SBP and DBP of kerosine and clean fuel users [23]. Of note is that the report by Wylie et al. is a secondary analysis; thus, the information used was not optimized for diagnosing gestational blood pressure [14]. For Arku et al., only information on cooking was collected from participants; using other fuels for other purposes, such as heating, was not considered [23].

Weber et al. [16] and Agrawal and Yamoto [25] investigated the effects of biomass and clean fuel exposure on obstetric outcomes and symptoms of pre-eclampsia/eclampsia, respectively. Weber et al. reported an increased likelihood of perinatal mortality and adverse 5 min Apgar score using biomass fuels [16]. At the same time, Agrawal and Yamoto found significant associations between the use of polluting fuels and symptoms of pre-eclampsia/eclampsia [25]. Case ascertainment was based on self-reported data rather than clinical assessment for these two studies.

Lee et al. [24] and Qu et al. [27] assessed the effects of biomass fuels and clean fuels on the symptoms of hypertension (HTN), coronary heart disease (CHD), and diabetes. The results of both studies indicated that biomass fuel is significantly associated with increased risk for all three disorders. In addition, Qu et al. reported the same observation for stroke and dyslipidemia [27]. One important thing to note is that both studies based their analyses on self-reported data, which can be subjected to recall bias.

Bassig et al. investigated the associations between exposure to biomass and clean fuel and ischemic heart disease (IHD) risk [22]. The findings indicated that biomass fuel was associated with an increased risk of IHD compared to clean fuel use. The authors did not collect information relevant to the etiological study of IHD, placing some restrictions on the results.

Both Yu et al. [17] and Mitter et al. [19] reported an increased likelihood of all-cause mortality and CVD mortality for biomass fuel users compared to clean fuel users. However, Mitter et al. lacked a control group for fuel exposure [19], while both studies used self-reported data for analysis. In the same vein, three studies [18,20,21] all reported an increased likelihood of CVD death due to exposure to biomass fuels compared to clean fuels. One was a cross-sectional study [21], while the remaining two were prospective cohort studies [18,20]. Again, all these studies based their analyses on self-reported data from the participants.

### 3.4. Patients’ Demographic Characteristics

The selected 16 papers encompassed 547,463 cases (Table 1). Among them, 319,180 cases were exposed to household air pollutants (HAP), whereas 228,283 were not. The average age of the included candidates ranged from 27.8 to 60 and 28.5 to 57 years among those exposed and those not exposed to HAP, respectively. Within females, 64.7% (254,426/393,502) were exposed to HAP, while 35.3% (139,076/393,502) were non-exposed. The smoking status was reported among 188,223 cases; 72.6% (136,597/188,223) were also exposed to HAP, while 27.4% (516,26/188,223) were not.

### 3.5. Association between HAP and Cardiovascular Diseases

#### 3.5.1. Association with CVD-Related Mortality

**Figure 2:**
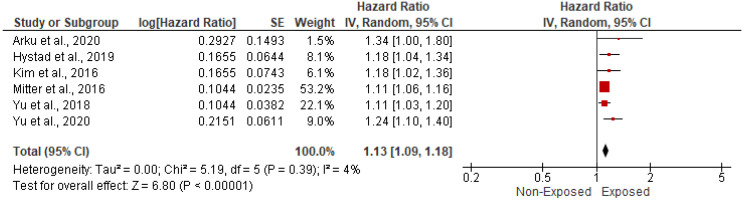
Forest plot of summary analysis of the hazard ratio and 95% CI of the association between HAP and CVD-related mortality. The size of the red squares is proportional to the statistical weight of each trial. The gray diamond represents the pooled point estimate. The positioning of both diamonds and squares (along with 95% CIs) beyond the vertical line (unit value) suggests a significant outcome (IV = inverse variance).

**Figure 3:**
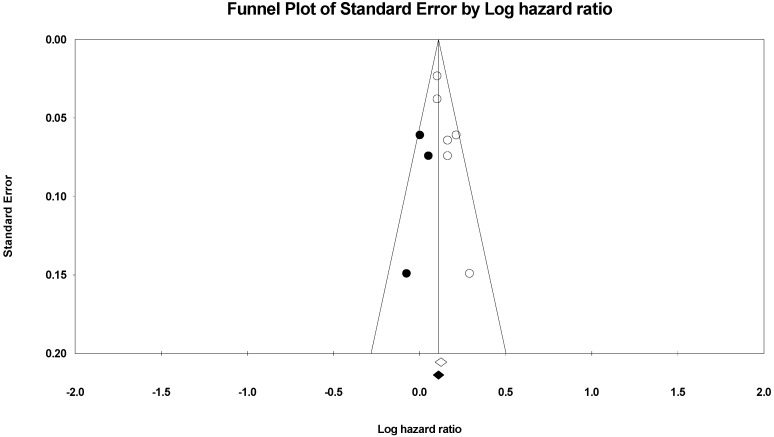
After using the trim and fill method of Duvall and Tweedie, a funnel plot of the observed and plotted studies for assessing the association between HAP and CVD-related mortality. The white circles represent the observed studies, and the black circles represent the plotted studies.

**Figure 4:**
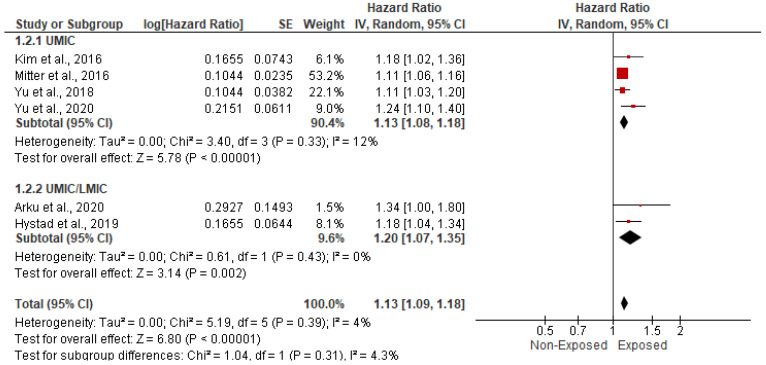
Forest plot of subgroup analysis of the hazard ratio and 95% CI of the association between HAP and CVD-related mortality based on income category. The size of the red squares is proportional to the statistical weight of each trial. The gray diamond represents the pooled point estimate. The positioning of both diamonds and squares (along with 95% CIs) beyond the vertical line (unit value) suggests a significant outcome (IV = inverse variance).

**Figure 5:**
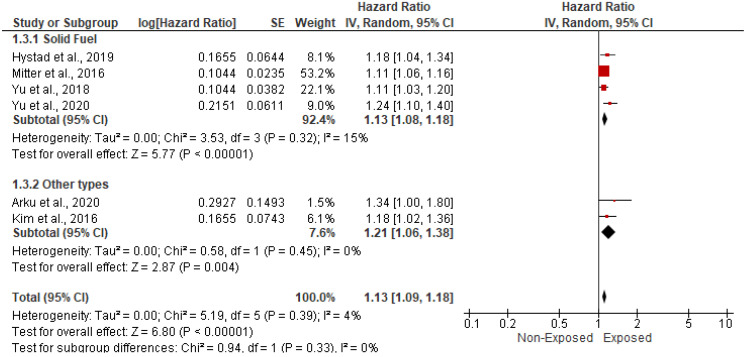
Forest plot of subgroup analysis of the hazard ratio and 95% CI of the association between HAP and CVD-related mortality based on types of HAP. The size of the red squares is proportional to the statistical weight of each trial. The gray diamond represents the pooled point estimate. The positioning of both diamonds and squares (along with 95% CIs) beyond the vertical line (unit value) suggests a significant outcome (IV = inverse variance).

The association between HAP and CVD-related mortality was assessed among 466,498 cases within six papers. In the random-effects model (*I*^2^ = 4%, *p* = 0.39), patients exposed to HAP were 13% more likely to die of cardiovascular diseases (HR = 1.13; 95% CI = 1.09–1.18; *p* < 0.001) (Figure 2). A significant publication bias was observed based on the asymmetrical distribution of studies along the funnel plot’s null line and on Egger’s regression test (intercept = 1.65, *p* = 0.019). This publication bias did not alter the significance of the yielded evidence (Figure 3). Subgroup analysis based on income category revealed a statistically significant impact of HAP on CVD-related mortality in UMIC (HR = 1.13; 95% CI = 1.08–1.18; *p* < 0.001) and UMIC/LMIC (HR = 1.20; 95% CI = 1.07–1.35; *p* = 0.002) (Figure 4). In this respect, subgroup analysis based on pollutants type revealed a statistically significant association between solid fuel HAP (HR = 1.13; 95% CI = 1.08–1.18; *p* < 0.001), other types of HAP (HR = 1.21; 95% CI = 1.06–1.38; *p* = 0.004), and CVD-related mortality (Figure 5).

#### 3.5.2. Association with Stroke and Cerebrovascular Accidents

**Figure 6:**
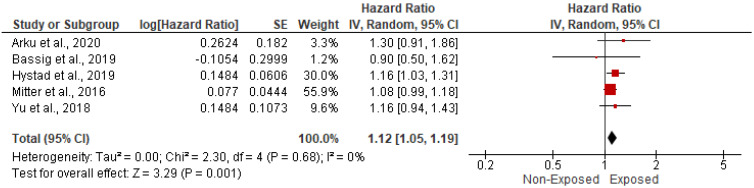
Forest plot of summary analysis of the hazard ratio and 95% CI of the association between HAP and stroke and cerebrovascular accidents. The size of the red squares is proportional to the statistical weight of each trial. The gray diamond represents the pooled point estimate. The positioning of both diamonds and squares (along with 95% CIs) beyond the vertical line (unit value) suggests a significant outcome (IV = inverse variance).

**Figure 7:**
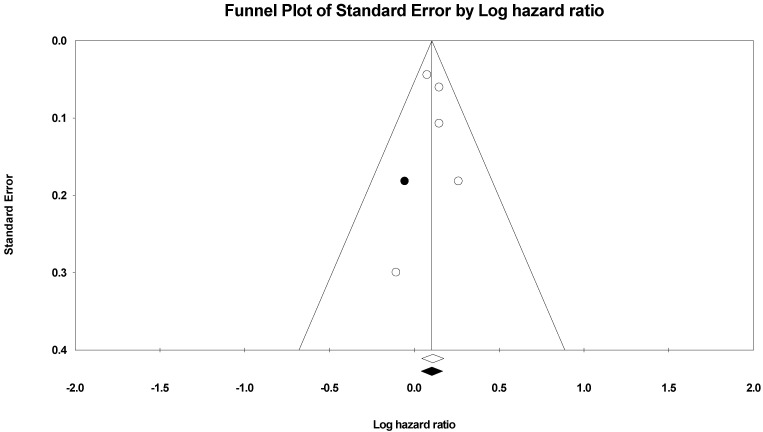
Funnel plot of the observed and plotted studies for assessing the association between HAP and cerebrovascular accidents after using the trim and fill method of Duvall and Tweedie. The white circles represent the observed studies, and the black circles represent the plotted studies.

**Figure 8:**
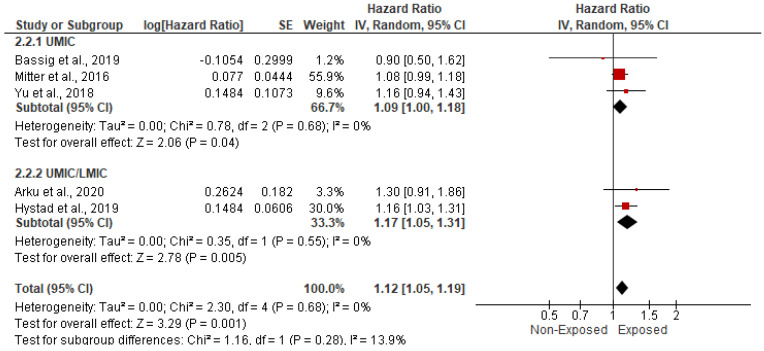
Forest plot of subgroup analysis of the hazard ratio and 95% CI of the association between HAP and cerebrovascular accidents based on income category. The size of the red squares is proportional to the statistical weight of each trial. The gray diamond represents the pooled point estimate. The positioning of both diamonds and squares (along with 95% CIs) beyond the vertical line (unit value) suggests a significant outcome (IV = inverse variance).

**Figure 9:**
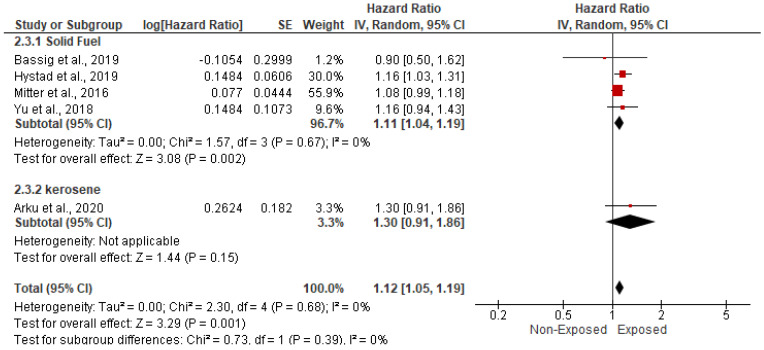
Forest plot of subgroup analysis of the hazard ratio and 95% CI of the association between HAP and cerebrovascular accidents based on types of HAP. The size of the red squares is proportional to the statistical weight of each trial. The gray diamond represents the pooled point estimate. The positioning of both diamonds and squares (along with 95% CIs) beyond the vertical line (unit value) suggests a significant outcome (IV = inverse variance).

Five studies, spanning 315,111 cases, assessed the impact of HAP on the risk of stroke and cerebrovascular accidents. In the random-effects model (*I*^2^ = 0%; *p* = 0.68), patients exposed to HAP were 12% more likely to die from cardiovascular disease (HR = 1.12; 95% CI = 1.05–1.19; *p* = 0.001) (Figure 6). There was no evidence of publication bias based on the symmetrical distribution of studies along the funnel plot’s null line and Egger’s regression test (intercept = 0.29, *p* = 0.7) (Figure 7). Subgroup analysis based on income category revealed a statistically significant impact of HAP on stroke and cerebrovascular accidents in UMIC (HR = 1.09; 95% CI = 1.00–1.18; *p* = 0.04) and UMIC/LMIC (HR = 1.17; 95% CI = 1.05–1.31; *p* = 0.005) (Figure 8). In this respect, subgroup analysis based on pollutants type did reveal a statistically significant association between solid fuel HAP (HR = 1.11; 95% CI = 1.04–1.19; *p* = 0.002) and stroke and cerebrovascular accidents. However, there was no statistically significant association between kerosene and stroke and cerebrovascular accidents (HR = 1.3; 95% CI = 0.91–1.86; *p* = 0.15) (Figure 9).

#### 3.5.3. Association with Myocardial Infarction

**Figure 10:**
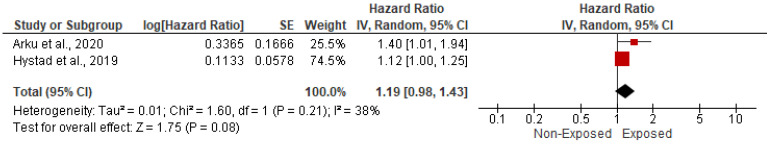
Forest plot of summary analysis of the hazard ratio and 95% CI of the association between HAP and myocardial infarction risk. The size of the red squares is proportional to the statistical weight of each trial. The gray diamond represents the pooled point estimate. The positioning of both diamonds and squares (along with 95% CIs) beyond the vertical line (unit value) suggests a significant outcome (IV = inverse variance).

The impact of HAP on the risk of myocardial infarction was assessed among 122,840 cases within two studies. In the random-effects model (*I*^2^ = 38%; *p* = 0.21), there was no statistically significant impact of HAP on the risk of myocardial infarction (HR = 1.19; 95% CI = 0.98–1.43; *p* = 0.08) (Figure 10).

#### 3.5.4. Association with Ischemic Heart Disease

**Figure 11:**
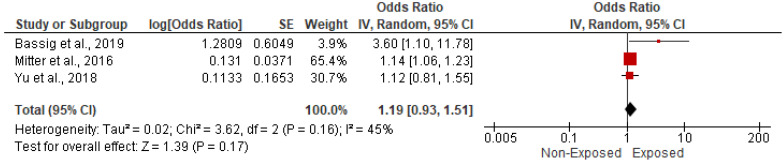
Forest plot of summary analysis of the Odds ratio and 95% CI of the association between HAP and ischemic heart disease. The size of the red squares is proportional to the statistical weight of each trial. The gray diamond represents the pooled point estimate. The positioning of both diamonds and squares (along with 95% CIs) beyond the vertical line (unit value) suggests a significant outcome (IV = inverse variance).

Three studies evaluated the impact of HP on the risk of ischemic heart disease among 192,271 candidates. In the random-effects model (*I*^2^ = 45%, *p* = 0.16), there was no statistically significant impact of HAP on the risk of myocardial infarction (OR = 1.19; 95% CI = 0.93–1.51; *p* = 0.17) (Figure 11).

#### 3.5.5. Association with Heart Failure

**Figure 12:**
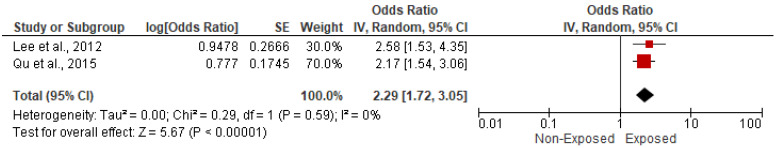
Forest plot of summary analysis of the Odds ratio and 95% CI of the association between HAP and heart failure. The size of the red squares is proportional to the statistical weight of each trial. The gray diamond represents the pooled point estimate. The positioning of both diamonds and squares (along with 95% CIs) beyond the vertical line (unit value) suggests a significant outcome (IV = inverse variance).

The risk of heart failure among patients exposed to HAP was assessed among 27,945 cases within two studies. In the random-effects model (*I*^2^ = 0%; *p* = 0.59), patients exposed to HAP were 2.3 times more susceptible to develop heart failure, in contrast to non-exposed cases (OR = 2.29; 95% CI = 1.72–3.05; *p* = 0.17) (Figure 12).

#### 3.5.6. Association with Hypertension

**Figure 13:**
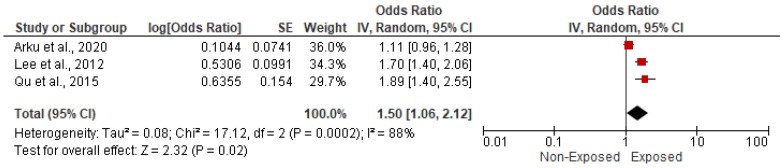
Forest plot of summary analysis of the Odds ratio and 95% CI of the association between HAP and hypertension. The size of the red squares is proportional to the statistical weight of each trial. The gray diamond represents the pooled point estimate. The positioning of both diamonds and squares (along with 95% CIs) beyond the vertical line (unit value) suggests a significant outcome (IV = inverse variance).

The risk of hypertension among patients exposed to HAP was assessed among 59,435 cases within three studies. In the random-effects model (*I*^2^ = 88%; *p* = 0.0002), patients exposed to HAP were 1.5 times more likely to be hypertensive, in contrast to non-exposed cases (OR = 1.5; 95% CI 1.06–2.12; *p* = 0.02) (Figure 13).

#### 3.5.7. Association with Eclampsia/Pre-Eclampsia

**Figure 14:**
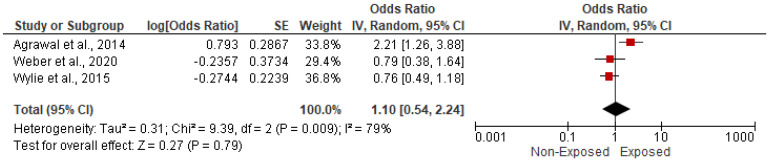
Forest plot of summary analysis of the Odds ratio and 95% CI of the association between HAP and eclampsia/pre-eclampsia. The size of the red squares is proportional to the statistical weight of each trial. The gray diamond represents the pooled point estimate. The positioning of both diamonds and squares (along with 95% CIs) beyond the vertical line (unit value) suggests a significant outcome (IV = inverse variance).

Three studies, which included 38,315 cases, reported the risk of eclampsia or pre-eclampsia among patients exposed to HAP. There was no statistically significant impact of HAP on the risk of eclampsia or pre-eclampsia (OR = 1.1; 95% CI = 0.54–2.22; *p* = 0.79) in the random-effects model (*I*^2^ = 79%, *p* = 0.009) (Figure 14).

#### 3.5.8. Association with Carotid Intimal Myocardial Thickness (CIMT)

**Figure 15:**
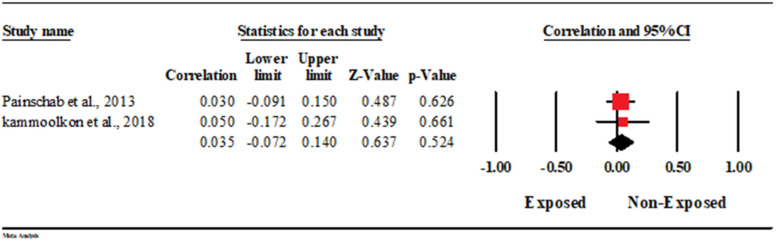
Forest plot of summary analysis of the correlation coefficient and 95% CI of the association between HAP and CIMT. The size of the red squares is proportional to the statistical weight of each trial. The gray diamond represents the pooled point estimate. The positioning of both diamonds and squares (along with 95% CIs) beyond the vertical line (unit value) suggests a significant outcome (IV = inverse variance).

The association between HAP and CIMT was reported in two studies that included 346 cases. In the random-effects model (*I*^2^ = 0%; *p* = 0.877), there was no statistically significant association between HAP and CIMT (Correlation = 0.035; 95% CI = −0.072–0.14; *p* = 0.524) (Figure 15).

## 4. Discussion

This systematic review indicates that there is limited literature investigating the impacts of household air pollutants from biomass fuels on the risk of developing cardiovascular diseases in low- and middle-income countries. However, such studies are increasing in number. The widespread use of biomass fuels for cooking and heating in LMICs is driving an increased rate of cardiovascular diseases. Thus, the results obtained herein can be leveraged to improve the quality of public health in the affected areas in terms of developing appropriate interventions. In the literature search stage, a systematic search was performed for all studies that were relevant to the research question and, at the same time, met all the specified inclusion criteria. Although the retrieved studies were not subjected to quality scoring using a pre-specified strategy, for each study found, the inherent weak points and limitations were thoroughly scrutinized to determine their impacts on the interpretations of the results obtained in each study. The evidence base for the chosen topic was limited. Several limitations were considered when interpreting the results obtained from this review. First, 9 of the 16 studies in this review were cross-sectional and therefore did not establish a temporal relationship between household air pollution and adverse cardiovascular health outcomes. The pooled relative risk (RR) estimates in a sensitivity analysis restricted to only longitudinal studies were attenuated.

Furthermore, access to individual participant-level data was unavailable, and therefore homogeneity of risk across all study participants for the health outcomes of interest had to be assumed. In addition, some of the studies included in the meta-analysis were observations with varying levels of adjustments. Finally, the studies were quite heterogeneous regarding the exposures compared, the assessed outcomes, and the potential for bias.

The techniques used to group exposure in the studies varied widely; while some studies classified exposure according to the type of fuels used, some classified them based on the period of use. In addition, some studies examined risks based on exposure to specific air pollutants. At the same time, some introduced various interventions to compare the impacts of exposure to fuels. For users of biomass fuels, there are bound to be significant differences in personal exposure to HAP. Such individual differences are driven by factors such as the year of use, the number of hours spent cooking per day, the primary and secondary fuels used by an individual, the design of the room where the cooking is done, and the design of the stove used for cooking. Primarily, the reviewed studies classified exposure based on biomass fuel use; thus, the estimated risks presented represent only rough means. On the other hand, it was not feasible for the studies to measure direct exposure to HAP. This method would be misleading because of variations in pollutant levels over time and differences in the exposure period.

Out of the sixteen selected studies, nine assessed the associations between the use of biomass fuels and various cardiovascular diseases. Three studies (Painschab et al., Kammoolkon et al., Tiwana et al.) were of cross-sectional design and had relatively small samples [13,15,28]. In contrast to the other two papers, which examined exposure to biomass fuels, Kammoolkon et al. examined the effects of exposure to incense smoke [15]. All three studies reported significant associations between biomass fuel (or incense) and cardiovascular conditions. Specifically, Painschab et al. reported that the mean CIMT (0.66 mm) for biomass fuel users is higher than that of clean fuel users (0.60 mm) [13]. In addition, carotid artery atherosclerotic plague (CAAP) is more prevalent in biomass fuel users than in clean fuel users. Similar results were obtained by Kammoolkon et al. [15]; this study reported that the daily incense users had a higher mean CIMT (0.75 ± 0.18 mm) compared to non-users, whose mean CIMT was (0.66 ± 0.10 mm). These findings are in line with the report of Wu et al., who found a significant positive relationship between the use of biomass fuel and an increased risk of CIMT and atherosclerotic plaques [29]. In contrast, Buturak et al. reported no significant positive associations between chronic biomass smoke exposure and CIMT [30]. In addition to their findings on CIMT, Painschab et al. reported that biomass fuel users are more likely to develop high blood pressure than clean fuel users [13]. Similarly, Tiwana et al. reported an increased risk of high blood pressure for polluting fuel users [28].

Three other studies out of these nine were prospective cohort studies [17,19,28]. Two, Mitter et al. [19] and Yu et al., 2018 [20], used relatively small samples compared to Yu et al. (2020) [20]. All three studies reported positive associations between biomass fuel use and increased likelihood of CVD mortality. For the increased likelihood of CVD mortality, the hazard ratio recorded by Mitter et al. was 1.11 [19], while Yu et al. (2018) [17] and Yu et al. (2020) [20] reported hazard ratios of 1.20 and 1.24, respectively.

The last three studies in this group were cross-sectional research with medium-sized samples that ranged from 13,877 to 16,325 participants [22,24,27]. Lee et al. [24] and Qu et al. [27] reported significant positive associations between exposure to smoke from biomass fuels and increased likelihood of hypertension (HTN), coronary heart disease (CHD), and stroke. Lee et al. reported hazard ratios of 1.70, 2.58, and 1.87 for HTN, CHD, and stroke, respectively [24], while Qu et al. reported hazard ratios of 1.751, 2.251, and 1.642 for the same disease conditions [27].

Four of the seven remaining studies investigated the impacts of biomass fuel use on CVD mortality [18,21,23,26]. Two, Hystad et al. [19] and James et al. [26], used a cross-sectional design, while the remaining two were prospective cohort studies. James et al. [26] had a relatively small sample size compared to the other three. Yet all four found positive associations between indoor exposure to smoke from biomass fuels and CVD mortality. Arku et al. [23] and Hystad et al. [21] recorded hazard ratios of 1.34 and 1.08 for CVD mortality, while James et al. recorded an odds ratio of 6.07 for the same condition [26]. Kim et al. reported that the risk of CVD mortality increases with increasing periods of coal use [18]. Of importance is that these studies assessed the cause of death by verbal autopsy. Although this validated the death, the potential for total accuracy is low. In addition to CVD mortality, Kim et al. investigated the associations between the use of biomass fuel and the risk of ischemic heart disease, myocardial infarction, and stroke [18]. This study found significant associations with an increased likelihood (HR = 1.61 and HR = 1.80) for ischemic heart disease and myocardial infarction, respectively. However, the use of biomass fuel (coal) was not associated with stroke mortality [18].

The final three studies assessed the impacts of biomass fuel exposure on obstetric outcomes in pregnant women [14,24,25]. While Agrawal and Yamoto examined the effects of biomass fuel exposure on the risk of developing pre-eclampsia/eclampsia [14] are potentially hazardous pregnancy complications characterized by hypertension, Wylie et al. assessed the effects of biomass fuel use on the risk of developing gestational hypertension [14]. Agrawal and Yamoto reported that women using biomass and solid fuels are more likely to develop pre-eclampsia/eclampsia symptoms [25]. The risk can be further increased by anemia, asthma, twin pregnancy, and abortion. In contrast, Wylie et al. found that pregnant women using biomass fuel have lower systolic blood pressure (BDP) and diastolic blood pressure (DBP) than those using clean fuels [14]. Furthermore, this study reported that hypertension was lower for pregnant women cooking with biomass fuels (14.6%) compared to clean fuel users (19.6%), but the value did not reach significance after adjustment [14]. Weber et al. examined hypertensive disorders of pregnancy and other obstetric outcomes (perinatal mortality, miscarriages, pre-term birth, low Apgar score one minute and five minutes after birth, postpartum hemorrhage, low birth weight, cesarean section, and small for gestational age) in pregnant women using biomass and clean fuels [16]. Apart from perinatal mortality and Apgar score at 5 min, all other outcomes, including hypertensive disorders, were negatively associated with biomass fuel use. Using polluting fuels had a positive relationship with perinatal mortality (OR = 7.6) and adverse Apgar score (<7) at 5 min (OR = 3.83) compared with clean fuel users. Qu et al. (2015) was the only study that examined the associations between biomass fuel use and dyslipidemia. That study found that polluting fuel was associated with an increased risk of dyslipidemia (OR = 1.185) [27].

Overall, there is substantial evidence for positive associations between HAP from biomass fuel use and an increased risk of hypertension (HTN), a recognized risk factor for cardiovascular diseases. From previous research, six studies [31,32,33,34,35,36] found a significant association between HAP and HTN, while one study [37] found no positive relationship between the two parameters. Furthermore, several studies reported significant positive relationships between exposure to HAP from biomass fuels and increased risk of various cardiovascular events. Brook et al. reported that exposure to HAP is an established trigger for cardiovascular events [38], while Al-Shammari reported significant associations between exposures to different types of biomass fuel and CVD [39].

## 5. Conclusions

The global burden of disease attributable to household air pollution is not evenly distributed worldwide, as it is higher in low- and middle-income countries. The current systematic review and meta-analysis suggest a positive association between exposure to household air pollution and cardiovascular outcomes; however, the evidence for several cardiovascular diseases is based on a very limited number of studies. There is also a lack of studies from Sub-Saharan Africa and South America, where solid fuel use for cooking and heating is high. However, there is still a need for additional research because the rate of cardiovascular diseases in LMICs is rising, and household air pollutants are part of the risk factors contributing to this trend. In addition, there are many inexpensive ways to reduce household air pollution from solid fuels. They were improving indoor air quality demands sustained and coordinated action at all levels. Low- and middle-income countries need to work together on solutions for sustainable, cleaner, and more inexpensive methods of cooking and heating, especially in rural areas. More timely and accurate studies like the current one are urgently needed, especially in LMICs, to facilitate the development of effective global health strategies that have the potential to curb the adverse health effects associated with household air pollution. There is also a need for evidence-based policy and decision-making to reduce the burden of cardiovascular disease related to household air pollution.

## Figures and Tables

**Table 1:** Demographic characteristics of the included articles.

Study ID		Study Regions	Income Category	Study Design	Study Period	Indoor Pollutants	Sample Size		Age		Gender (Male)		Gender (Female)		Body Mass Index		Completed Primary School		Comorbidities					
																			Cardiovascular Disease		Smoking		Alcohol Drinking	
							Exposed	Non-Exposed	Exposed	Non-Exposed	Exposed	Non-Exposed	Exposed	Non-Exposed	Exposed	Non-Exposed	Exposed	Non-Exposed	Exposed	Non-Exposed	Exposed	Non-Exposed	Exposed	Non-Exposed
							Number	Number	Mean ± SD	Mean ± SD	Number	Number	Number	Number	Mean ± SD	Mean ± SD	Number	Number	Number	Number	Number	Number	Number	Number
1	Painschab et al., 2013 [13]	Peru	UMIC	Cross-sectional study	February and October 2011	PM2.5	154	112	58 ± 12	55 ± 12	63	60	91	52	24 (22–27) *	27 (23–30) *	102	40	2	20	4	12	NR	NR
2	Wylie et al., 2015 [14]	India	LMIC	Cross-sectional study	December 2006 To May 2008	Wood and gas fuel	1134	235	NR	NR	0	0	1134	235	17	8	NR	NR	NR	NR	2	0	23	1
3	kammoolkon et al., 2018 [15]	Thailand	UMIC	Cross-sectional study	July to August 2016	Incense exposure	37	43	60 ± 10	57 ± 11.0	8	11	29	33	24.13 ± 3.22	23.46 ± 3.38	22	22	2	0	1	2	4	6
4	Weber et al., 2020 [16]	Ghana	LMIC	Prospective cohort study	July 2012 to March 2014	Wood, charcoal, crop residue, and kerosene	279	540	27.8 ± 5.6	28.5 ± 4.8	0	0	279	540	25.0 ± 4.6	25.8 ± 4.8	44	206	NR	NR	NR	NR	NR	NR
5	Yu et al., 2018 [17]	China	UMIC	Prospective cohort study	June 2004 and January 1, 2014	Solid fuel exposure	150,992	26,559	53.13 ± 10.09	48.2 ± 9.6	29,231	7274	121,761	19,285	23.11 ± 3.36	23.7 ± 3.2	11,194	5498	NR	NR	114,228	18,857	48,496	10,199
6	Kim et al., 2016 [18]	China	UMIC	Prospective cohort study	1996 and December 2009	Household coal	46,287	27,076	52.05 ± 9.16	51.97 ± 8.88	0	0	46,287	27,076	24.13 ± 3.46	23.81 ± 3.35	12,113	8377	3350	2003	1429	614	1104	546
7	Mitter et al., 2016 [19]	Iran	UMIC	Prospective cohort study	2004–2008	Household fuels	1578	NR	NR	NR	NR	NR	NR	NR	NR	NR	NR	NR	NR	NR	NR	NR	NR	NR
8	Yu et al., 2020 [20]	China	UMIC	Prospective cohort study	From baseline until December 31, 2016	Solid fuel exposure	15,381	75,785	57.1 (10.6) *	49.2 (10.1) *	4049	28,708	11,332	47,077	22.9 ± 3.4	24.4 ± 3.4	979	38,047	NR	NR	3049	20,605	4257	48,213
9	Hystad et al., 2019 [21]	Bangladesh, Brazil, Chile, China, Colombia, India, Pakistan, Philippines, South Africa, Tanzania, and Zimbabwe	UMIC/ LMIC	Cross-sectional study	2002 to 2015	Solid fuel exposure for cooking	38,187	53,163	NR	NR	15,883	21,709	22,304	31,454	NR	NR	25,031	12,754	2984	8542	9965	10,283	7269	10,630
10	Bassig et al., 2019 [22]	China	UMIC	Cross-sectional study	1 January 1976 to 31 December 2011	Solid fuel exposure for cooking	11,188	1954	NR	NR	0	0	11,188	1954	NR	NR	2600	286	NR	NR	NR	NR	NR	NR
11	Arku et al., 2020 [23]	India, China, South Africa, Tanzania	UMIC/ LMIC	Prospective cohort study	2001 and 2018	Kerosene for cooking	2767	28,723	NR	NR	2639	28,592	128	131	NR	NR	NR	NR	17	25	66	50	60	44
12	Lee et al., 2012 [24]	China	UMIC	Cross-sectional study	August 2007 to July 2009	Kerosene for cooking	11,013	3055	NR	NR	5053	1410	5960	1645	NR	NR	4502	1562	NR	NR	2717	498	NR	NR
13	Agrawal et al., 2014 [25]	India	LMIC	Cross-sectional study	2005–2006	Cooking Smoke	28,158	7969	NR	NR	0	0	28158	7969	NR	NR	12,959		NR	NR	608		911	
14	James et al., 2020 [26]	India	LMIC	Cross-sectional study	August 2016 and September 2018	Wood, crop residues, animal dung, and charcoal	566	582	NR	NR	NR	NR	NR	NR	NR	NR	NR	NR	NR	NR	NR	NR	NR	NR
15	Qu et al., 2015 [27]	China	UMIC	Cross-sectional study	2010–2012	Coal, wood fuel, and straw	11,390	2487	NR	NR	5615	826	5775	1625	NR	NR	NR	NR	NR	NR	5120	749	6947	1720
16	Tiwna et al., 2020 [28]	Nepal	LMIC	Case–control study	NR	Wood, biogas, and LPG	69	35	NR	NR	NR	NR	NR	NR	NR	NR	4	4	NR	NR	16	6	10	4

**Abbreviations: UMIC** = upper middle-income countries, **LMIC** = lower middle-income countries, **LPG =** liquefied petroleum gas, **SD** = standard deviation, **NR** = non-reported, * median and range.

## Data Availability

Extracted data can be made available on request.
